# Potential Micronutrient Deficiencies in the First 1000 Days of Life: The Pediatrician on the Side of the Weakest

**DOI:** 10.1007/s13679-024-00554-3

**Published:** 2024-03-21

**Authors:** Carolà Panzeri, Luca Pecoraro, Alice Dianin, Andrea Sboarina, Olivia C. Arnone, Giorgio Piacentini, Angelo Pietrobelli

**Affiliations:** 1 Department of Surgical Sciences, Dentistry, Gynecology and Pediatrics, Pediatric Clinic, University of Verona, P.Le Stefani, 1 – 37126, Verona, Italy; 2 Regional Centre for Newborn Screening, Diagnosis and Treatment of Inherited Metabolic Diseases and Congenital Endocrine Diseases, Pediatric Clinic, Azienda Ospedaliera Universitaria Integrata, Verona, Italy; 3 Department of Surgical Sciences, Dentistry, Gynecology and Pediatrics, University of Verona, P.Le Stefani, 1 – 37126, Verona, Italy; 4 Pennington Biomedical Research Center, Baton Rouge, LA 70808 USA

**Keywords:** Micronutrients, 1000 days, Vegetarian diet, Exclusion diet, Insufficient micronutrients storage, Micronutrients adsorption defect

## Abstract

**Purpose of Review:**

This study is to examine potential micronutrient deficiencies and any need for supplementation in children following specific diet plans in the first 1000 days of life.

**Recent Findings:**

Optimal nutrition in the first 1000 days of life has a lifelong positive impact on child development. Specific intrauterine and perinatal factors, pathological conditions, and dietary restrictions can represent potential risk factors for micronutrient deficiencies in the first 1000 days of life, which can have negative systemic consequences. Preterm and low-birth-weight infants are intrinsically at risk because of immature body systems. Children affected by cystic fibrosis are prone to malnutrition because of intestinal malabsorption. The risk of micronutrient deficiency can increase in various situations, including but not limited to children following selective dietary regimens (vegetarian and vegan diets and children affected by specific neuropsychiatric conditions) or specific dietary therapies (children affected by food allergies or specific metabolic disorders and children following restricted diet as a part of therapeutic approach, i.e., ketogenic diet for epilepsy). In light of this situation, the micronutrient status in these categories of children should be investigated in order to tailor strategies specific to the individual’s metabolic needs, with a particular focus on deficiencies which can impair or delay the physical and cognitive development of children, namely, vitamin B12, vitamin D and folic acid, as well as oligo-elements such as iron, zinc, calcium, sodium, magnesium, and phosphorus, and essential fatty acids such as omega-3.

**Summary:**

Identification of micronutrient deficiency in the first 1000 days of life and timely supplementation proves essential to prevent their long-term consequences.

**Graphical Abstract:**

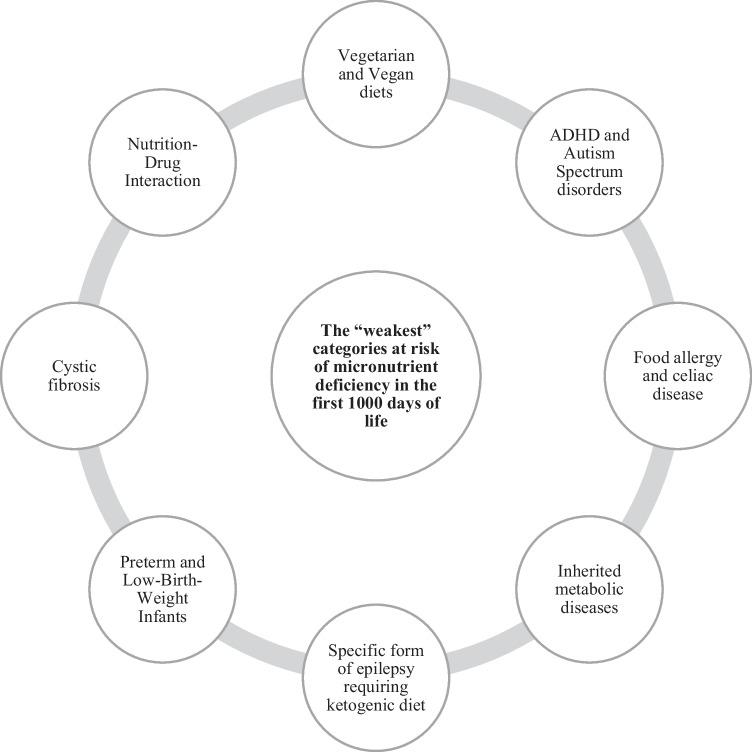

**Supplementary Information:**

The online version contains supplementary material available at 10.1007/s13679-024-00554-3.

## Introduction

The first 1000 days (conception, pregnancy, and the first two years of life) represent a period of enormous vulnerability to nutritional deficiency as well opportunity for promoting better outcomes in children’s lives. The fetuses, infants, and children experience unique physiological changes and have specific nutritional needs [1]. In this context, an optimal nutrition during this time acts as the first line of prevention against developmental shortfalls [2–6]. The traditional concept of optimal nutrition during the first 1000 days of life included only macronutrient and energy balance. This concept was recently extended to include an adequate supplementation of micronutrients, given the evidence that diet influences gene expression through epigenetic mechanisms during the first 1000 days of life. Adequate micronutrient intake is essential for neural, visual, and skeletal system development because of its role in early fetal organ development and cell differentiation [7, 8•, 9•, 10–20, 21•, 22–26]. Micronutrient can only be provided by the diet and act as coenzymes in the production of hormones and essential substances for proper growth. Hence, their role in phases of rapid growth as the first 1000 days is essential. There is no definitive evidence on the benefits of supplementation of other micronutrients without demonstrated deficiency, both for lactating mothers and both for breastfed and weaning infants. At the same time, a supplementation of micronutrients become necessary during the first 1000 days of life in infants or children adopting restricted or unbalanced diets and affected by particular diseases [16, 27, 28••]. In fact, infants or children affected by specific intrauterine and perinatal factors or pathological conditions, as well as preterm/low birth weight infants, could be particularly at risk of micronutrient deficiencies. This article explores potential early-life micronutrient deficiencies and their associated risks in the context of some of the most common chronic conditions during early childhood. Specifically, it aims to urge pediatricians to pay more attention to potential micronutrients deficiencies in these infants and children.

## Importance of Micronutrients During the First 1000 Days

Micronutrients at significant risk of deficiency during the first 1000 days are reported in Table 1. Among those, deficiencies of omega-3 fatty acids, vitamins C, B9, B12, and D, and minerals such as iodine and iron seem the most involved in clinical syndromes [1]. It is important to note that unbalanced diets can lead to micronutrient deficiencies even when following an omnivorous diet due to maternal and child undernutrition and food insecurity especially in low-income and middle-income countries. In this paragraph, we have focused on dietary regimens that involve the conscious choice to exclude food groups or the therapeutic necessity to do so.

**Table 1:** High risk categories of infants and children for micronutrient deficiency in the first 1000 days of life, modified from [IV revision of Dietary Reference Intake for the Italian Population (LARN 2014). Milan: SICS Editore]

**Micronutrients most involved in clinical syndromes during the first 1000 days of life**	**Main natural food sources of the micronutrient**	**Impacts of micronutrient deficiency and comorbidities in newborns and children**	**Diseases and diet plans prone to micronutrient deficiency**
**Vitamin B9 or folic acid**	Meat (especially beef liver), legumes, leafy greens and other vegetables (spinach, kale cabbage, arugula, asparagus, broccoli), oats, nuts and seeds, whole cereals	Anemia, hyperhomocystinemia, vascular and cardiac diseases, neural tube malformations [30]	Inadequate supplementation during pregnancy, gastrointestinal malabsorption, prolonged pharmacological use, genetic polymorphism, metabolic and ketogenic restrictive dietary therapies without supplementation
**Vitamin B12 or cobalamin**	Meat and organ meats, fish and shellfish (clams, oysters, crab), eggs, milk and dairy products	Megaloblastic anemia and neurological alterations, in worst cases, irreversible lesions of central nervous system. Early signs are irritability, failure to thrive, food refusal [14–16, 31•]	Not supplemented vegetarian diets, gastrointestinal malabsorption, prolonged pharmacological use, metabolic and ketogenic restrictive dietary therapies without supplementation
**Vitamin C**	Fruits (guava, kiwi, strawberry, oranges, papaya, mango, cantaloupe, berries, lemon, grapefruit), vegetables (bell peppers, tomatoes, broccoli, spinach, kale)	Irritability, scurvy, impaired wound healing, increased infection risk, bleeding gums, skin problems, fatigue, anemia [28••, 29••]	Diets with low vegetable and fruit intake, metabolic and ketogenic restrictive dietary therapies without supplementation
**Vitamin D**	Fatty fish (mackerel, herring, tuna, salmon, cod liver oil), butter, high fat hard cheese, eggs (yolk), mushrooms	Rickets, osteoporosis, impaired bone growth, muscle spasm and seizures, increased risk of cardiovascular and tumoral diseases [20, 21•, 22, 26]	Limited sun exposure, skin pigmentation, obesity, gastrointestinal malabsorption, antiepileptic drugs, metabolic and ketogenic restrictive dietary therapies without supplementation
**Iron**	Meat, fish, shellfish, legumes, tofu and tempeh, leafy greens, quinoa, nuts and seeds (pumpkin seeds, cashews), dried fruits (apricot, raisins)	Anemia, cognitive impairment, glossitis, cheilosis, increased risk of infections [7, 19, 20, 32]	Chronic blood loss (ulcers, colon polyps, heavy menstrual bleeding, malabsorption, unbalanced vegetarian diets, metabolic and ketogenic restrictive dietary therapies without supplementation
**Iodine**	Fish, seaweed, dairy products, iodized salts, eggs, shellfish	Hypothyroidism, goiter, cretinism, developmental delay, deaf-mutism short stature [7, 17, 18]	Unbalanced vegetarian diets, autoimmune thyroid conditions, metabolic and ketogenic restrictive dietary therapies without supplementation
**Omega 3 fatty acids**	Fatty fish (salmon, mackerel, trout, sardines, herring), seeds, and nuts (flaxseeds, chia, walnuts, hemp)	Cognitive impairment, depression and mood disorders, skin problems, impaired neuronal and eye development in fetus [30, 33, 34]	Unbalanced vegetarian diets, gastrointestinal malabsorption, metabolic and ketogenic restrictive dietary therapies without supplementation

Vitamin C promotes the absorption and conservation of iron and stimulates the body’s immune system because of its antioxidant and anti-infective properties. Newborns with vitamin C deficiency may be irritable and may not gain weight as expected. Bone growth is impaired in infants and children, and bleeding and anemia may occur. Infections may develop with difficult-to-heal wounds [28••, 29••].

Vitamin B9, or folic acid, promotes the embryo’s neural tube closure; therefore, its deficiency may cause neural tube malformations. Folate requirements increase during pregnancy due to fetal and maternal tissue growth and active transfer of folate to the fetus. The benefit of daily folic acid supplementation for all females is well demonstrated in the periconceptional period [9•]. Choline insufficiency can result in neural tube defects as well [30].

Vitamin B12, or cobalamin, is involved in erythropoiesis, the synthesis of DNA and fatty acids, energy production, and functioning of the nervous system. A deficit results in megaloblastic anemia and nervous system, mood, and memory disorders. Neurological symptoms include tingling in feet and hands, loss of sensation, and weakness in arms and legs. In infants with nutritional vitamin B12 deficiency, early clinical symptoms are irritability, failure to thrive, and food refusal, accompanied, in worst cases, by central nervous system lesions, which can be irreversible [14–16, 31•].

Vitamin D promotes the absorption of calcium and phosphorus and bone remodeling. Studies also show its anti-inflammatory, anti-tumoral, and cardiovascular protection effects [22]. In vitamin D deficiency, the body absorbs less calcium and phosphate, causing bone disorders associated with bone weakness (rickets in children or osteomalacia in adults). Vitamin D deficiency during pregnancy can cause deficiency in the fetus; sometimes, the deficiency is severe enough to cause osteomalacia in women. Muscle spasms (tetany) caused by a low calcium level in the blood in people may be newborns’ first signs of rickets when severe vitamin D deficiency occurs. If the spasms are severe, they can lead to seizures. In younger children with rickets, the entire skull may be soft. Infants may have difficulty sitting up, crawling, and learning to walk. Closing of fontanelles may take longer. In children older aged one year or more, bone growth may be impaired, resulting in an abnormal spine curvature (scoliosis) and varus or valgus knees. Vitamin D supplementation is advised in all infants for the first year with 400 UI/die, whether breast or formula-fed [20, 21•, 22, 26].

Iodine is mainly involved in the synthesis of thyroid hormones. Severe maternal iodine deficiency delays fetal growth and brain development, causing congenital hypothyroidism, including intellectual disability, deaf-mutism, difficulty walking, short stature, and sometimes hypothyroidism (cretinism) [7, 17, 18].

Iron deficiency develops in stages. First, the demand for iron exceeds the amount consumed in the diet, causing the progressive depletion of iron reserves in the bone marrow. When reserves are reduced, the absorption of dietary iron increases to compensate for this deficiency. The deficiency impairs red blood cell synthesis in later stages, ultimately causing anemia. Severe and prolonged iron deficiency can also cause a functional alteration of cellular enzymes that contain iron. Most iron deficiency symptoms are due to anemia. In addition, patients may suffer from pica, a compulsive desire to eat nonfood substances (e.g., ice, dirt, paint, starch, ash). Other severe deficiency symptoms include skin and mucosal damage, such as glossitis and cheilosis [7, 19, 20, 32].

Omega-3 s are polyunsaturated fatty acid (PUFA) that includes eicosapentaenoic acid (EPA) and docosahexaenoic acid (DHA), as well as the essential precursor alpha-linolenic acid (ALA). Omega-3 deficiency is associated with skin problems, mood disorders, and attention deficits and difficulty to concentrate. In the fetus, they act in neuronal and eye development [30, 33, 34].

## Different Diets at Risk of Micronutrient Deficiency

Food of plant origin contains higher amounts of carbohydrates and dietary fiber and lower amounts of saturated fats and proteins than food of animal origin. Due to the lower fat content, a diet containing predominantly plant-based foods provides, on average, lower daily energy intake and reduced meal energy density. The high-fiber intake may interfere with the absorption of some minerals, especially iron, zinc, and calcium, due to phytates present in cereals, seeds, and legumes [28••, 29••]. A lower consumption of fats can have some beneficial effects on BMI and cardiovascular health especially reducing saturated fat but, on the other hand, attention should be paid to the types and sources of fats, particularly regarding the risk of inadequate absorption of omega-3 fatty acids. Among macronutrients, a qualitatively and quantitatively adequate protein intake is essential for the correct growth of children. Moreover, essential amino acids cannot be synthesized from the human organism and are present in adequate concentration prevalently in food of animal origin. Therefore, plant-based foods often lack specific amino acids.  It is important to consume plant-based foods of different groups throughout the day in order to compensate for specific amino acid deficiencies.  Breastfed infants have a sufficient protein intake even if the mother follows a vegetarian diet, but are exposed to a risk of protein malnutrition and micronutrient deficiency when given plant-based non-dairy milk alternatives, such as cereal, legume or nut “milk”. Soy or plant-based formulas, enriched with necessary proteins and micronutrients, can instead be easily used [30, 31•]. Regarding micronutrients**, **they appear to be more represented, varied, and bioavailable in animal-source foods than in foods of plant origin. Hence, it can be assumed that vegetarian diets can represent a risk for infants and children’s health in their first 1000 days of life. Data currently available from conducted studies do not formally establish a clear relationship between these dietary patterns and the intake or status of micronutrients [28••].

## Vegetarian Diets

The degree of animal-source food restriction defines the different vegetarian patterns, even though a more comprehensive approach also considers the diversity of included foods, as illustrated in Table 2. Historically, vegetarian diets have always been discouraged during the first 1000 days of life due to concerns about nutritional deficiencies and poor growth. On the contrary, position papers from North America state that well-planned vegetarian and vegan diets, when appropriately supplemented, are suitable for all life stages [32]. European statements strongly recommend that vegetarian/vegan diets should not be adopted without medical and dietetic supervision [33, 34]. Micronutrient deficiency risk is present for all vegetarian diets and specifically includes micronutrients of which animal-source food represents a more bioavailable origin: B12, vitamin A, iron, zinc, vitamin D, and essential fatty acids. On the other hand, vegetarian diets do not expose children to copper or selenium deficiency risk. During pregnancy and breastfeeding, vegetarian diets provide sufficient protein and micronutrient amounts, except for B12 and iron, which the mother should supplement. The main nutritional deficit of breast milk of vegetarian mothers is related to vitamin B12, with the risk for micronutrient deficiency depending on how restrictive the diet is. The maternal vegetarian diet represents the main cause of vitamin B12 deficiency in breastfed infants [36]. Recommendations for supplementations of other micronutrients during pregnancy and breastfeeding vary among countries. It seems prudent to suggest personalized supplementation only under medical supervision.

**Table 2:** Details of principal models of vegetarian diets and associated potential micronutrient deficiencies

Definition	Excluded foods	Consumed foods	Micronutrient deficiency risk [7, 8•, 9•, 10–20, 21•, 22–26]
Lacto-ovo-vegetarians (LOV)	Meat and fish	Dairy, eggs, honey + plant food	Risk for B12, calcium, iron, zinc, vitamin D, essential fatty acids [9•, 10–12, 28••, 29••, 30, 31•, 32–35]
Lacto-vegetarians	Meat and fish + eggs	Dairy + plant food	With respect to LOV: higher risk for B12 and iron acids [9•, 10–12, 28••, 29••, 30, 31•, 32–35]
Ovo-vegetarians	Meat and fish + dairy	Eggs + plant food	With respect to LOV: higher risk for calcium, vit acids [9•, 10–12, 28••, 29••, 30, 31•, 32–35]
Vegans	All animal products (meat, fish, dairy, eggs and honey, and foods that use ingredients derived from the processing of muscle or dairy foods, such as gelatin and rennet)	Plant food	Very severe risk for B12 With respect to LOV: higher risk for calcium, vitamin D, iron, zinc, essential fatty acids Risk for vitamin A [35–37, 38••]
Macrobiotic diet	Varies from being strictly vegetarian to avoiding dairy, eggs and some vegetables to more liberal options, including fish in some cases		Greater risk than vegan diet [31•]
Raw food diet	Raw vegetables, fruits and seeds, milk, and eggs		Greater risk than vegan diet
Fruit diet	Fresh and dried fruits, seeds, and some vegetables		Greater risk than vegan diet
Ketogenic diet	Limited amount of carbohydrates		Vitamin B, vitamin C, vitamin D, calcium, selenium, and magnesium [42, 43]

### Lacto-ovo-vegetarian Diet

The lacto-ovo vegetarian diet is the most common type of vegetarian diet; it excludes meat and fish but includes dairy products and eggs. A particular category is represented by flexitarians, who occasionally eat meat or fish. Upon the beginning of complementary feeding, a lacto-ovo-vegetarian diet exposes infants to a low risk of micronutrient deficiency if it includes a sufficient intake of dairy products [28••, 31•]. Nonetheless, most plant-based foods have low iron and zinc content and their bioavailability from vegetarian sources is lower than from animal sources.  Dietary advice should be given regarding food preparation techniques due to the possible mineral leaching during cooking and regarding limitation of the fiber content of the diet in order to increase zinc and iron absorption [37]. Children are at risk of iron deficiency due to their rapid growth. Parallelly, infants following a vegetarian diet are particularly at risk for zinc deficiency and should be monitored. For the same reason, during the first 1000 days of life, children following a vegetarian diet should have a higher iron intake (1.8 × intake of omnivorous children) [34], which can be guaranteed in most cases only through supplementation. The recommended iron prophylaxis for full-term newborns is 1 mg/kilogram per day (maximum 15 mg/die) from birth; its importance is linked to the evidence that iron deficiency in the early years of life has been related to anxiety, depression, and schizophrenia in adult age. Although the risk of B12 deficiency for infants on a lacto-ovo-vegetarian diet is lower than for infants following more restrictive diets, supplementation is encouraged. Due to the lack of fish in the diet, the risk of insufficient intake of essential fatty acids should be taken into consideration, especially ALA (and α-linolenic acid); supplementation of 100–200 mg of DHA daily is suggested [38••].

### Lacto-vegetarian Diet

A lacto-vegetarian diet excludes eggs. As a consequence, children following a lacto-vegetarian diet have a higher risk of vitamin B12 and iron deficiency [35].

### Ovo-vegetarian Diet

The ovo-vegetarian diet excludes milk and dairy products. As a consequence, for children following an ovo-vegetarian diet, calcium and vitamin D supplements are required to meet recommended intakes. Furthermore, due to the increasing level of restriction of animal source food, the risk of vitamin B12 deficiency is higher than in lacto-ovo-vegetarian diet [35].

### Vegan Diet

The vegan diet is the most restrictive and excludes all animal source food, as well as products containing ingredients derived from animal source foods. The major issue in the vegan diet is the total lack of vitamin B12 in food of plant origin. Severe vitamin B12 deficiency in the first 1000 days of life has been associated with altered neuronal myelination, which causes damage to the auditory and visual systems e interferes with learning and social interaction [36]. Symptoms and long-term prognosis depend on the severity and duration of the deficiency. All mothers and infants who follow vegetarian or vegan diets should take supplements. The dosage for Vitamin B12 in infants is 0.4 μg/day for the first 6 months, increasing up to 5 μg/day until the age of 3 years [33]. Newborns with vitamin B12–deficient mothers are usually asymptomatic at birth and develop clinical signs at 4–6 months of age, ranging from megaloblastic anemia to irreversible neurological damage.

Vitamin A is present in foods of both animal and plant origin; its bioavailability is conditioned by the quantity and quality of lipids consumed, which enable its absorption. The risk of vitamin A deficiency is low for the lacto-ovo-vegetarian diet but increases for pre-school children following vegan diets due to their limited food preferences [28••] On the other hand, dietary intake has a limited influence on individual vitamin D status because few foods are rich in vitamin D, mostly foods of animal origin. Both lacto-ovo-vegetarian and vegan diets poorly guarantee optimal vitamin D intake, even if the risk of deficiency seems higher in children following a vegan diet. Vitamin D supplementation is required in the first year regardless of the diet followed (600 UI/die in pregnancy, 400UI/die in the first year of life, then dose adjusted according to dietary habits and sun exposure). Calcium deficit is infrequent in the lacto-ovo-vegetarian diet because of the consumption of dairy products. On the contrary, children who follow a vegan diet do not have sufficient calcium intake to cover their requirements. The calcium content of breast milk is not influenced by a vegan maternal diet; however, at the beginning of complementary feeding, it is necessary to ensure adequate calcium intake through fortified foods or supplements. The calcium requirement in the first two years of life is 120 mg/day and requires an intake of approximately 300 mg/die [35, 37, 38••].

The vegan diet also poses a higher risk of iron-deficiency compared to other vegetarian diets. Children following an ovo-vegetarian diet get a small quantity of heme–iron from eggs, which is more-bioavailable, while subjects who follow a vegan diet introduce only non-heme iron through their diet. Moreover, iron bioavailability is limited by dietary fiber and phytate but increased by vitamin C. Therefore, it is necessary to consume foods which are fortified with iron [28••].

Regarding long-term consequences on health, it is not possible to establish with certainty at what age the beginning of a vegetarian diet can come without side effects on growth and nutritional status [35]. Benefits of vegetarian and vegan diets include lower BMI and lower risk of obesity and hypertension, with reduced levels of lipids, lipoproteins, glucose, insulin, and C-reactive protein when compared to omnivorous diets [35, 39]. No significant differences were found regarding thyroid function, precocious puberty, premature thelarche, or menstrual irregularities [35]. No conclusive results are available regarding the relationship between dietary patterns and neoplastic risk; the effects of the vegetarian diets are likely due not only to the exclusion of meat but also to the inclusion of a wide range of plant foods containing potentially protective substances [35, 40]. The effects of nutrient deficiencies are strongly related to the stage of development of each specific brain area and tend to be irreversible. Considering the important short and long-term outcomes of nutrient deficiency on neurodevelopment, vegan diets in the first 1000 days without proper supplementation are considered inadequate for optimal psychomotor development [35].

### Macrobiotic Diet

Macrobiotic diets are even more restrictive than vegan diet and are based on cereals, seeds, vegetables, seaweed, and soy products and may include fish. Children following macrobiotic diets present a similar profile of risk for micronutrient deficiency similar to that of children on vegan diet, with the main risk being vitamin B12 deficiency. Based on reported cases, restrictive diets such as a macrobiotic diet are inappropriate during a child’s early development because they have a negative impact on growth and micronutrients status [31•].

## Medical Conditions Associated with Micronutrient Deficiencies

### Genetic Conditions

#### Inherited Metabolic Diseases

It is known that diet represents a primary form of treatment for many inherited metabolic diseases and is essential to prevent and minimize intellectual disability and epilepsy. Therapeutic strategies can include protein-restricted diet, single or multiple amino acids-restricted diet, ketogenic diet, fat-restricted diet, galactose free diet, fructose-free diet, and targeted supplementation of macro and micronutrients. Moreover, nutrition plays a crucial role in sick-day emergency protocols, especially in younger children with organic acidurias, certain aminoacidopathies, urea cycle disorders, and fatty acid oxidation defects [41]. The restriction of specific diet components potentially reduces micronutrient status; hence, a comprehensive vitamin and mineral supplement should be provided and becomes an essential adjunct to dietary treatment. Moreover, feeding differences are common in children with inherited metabolic disorders, with meals complicated by poor appetite, limited food variety, and lengthy mealtimes [41–43]. Phenylketonuria (PKU) is the most common inherited disorder of amino acid metabolism. It results from deficiency in phenylalanine hydroxylase. Newborn screening for PKU facilitates the beginning of treatment right after birth and helps prevent intellectual disability, mental health disorders, and major health problems. The basis of the treatment in PKU patients is through diet: They need to follow a low-phenylalanine diet, although newer medications may allow some people with PKU to eat a diet that has a higher or an unrestricted amount of phenylalanine depending on their residual enzyme activity [44]. Treatment consists of three parts: natural protein or phenylalanine restriction to 25% or less of what would be a regular intake, phenylalanine-free L-amino acid supplements to meet protein and other nutrients requirements, and low protein food to meet energy requirements [45••]. In fact, a source of L-amino acids is required, and supplementation in PKU patients is achieved through L-amino acid supplements with added age-specific vitamin and mineral profiles, including pregnancy. No tailored micronutrient dietary reference values have been established for PKU, so the optimal intake of micronutrients on a low phenylalanine diet is unknown [44, 46]. Guidelines recommend to supplement micronutrients according to the daily requirements for healthy people. Most L-amino acid supplements have added vitamins and minerals; therefore, taking any additional supplements may not be necessary to meet the daily nutrient requirements of PKU patients [47]. Amino acid supplements are not palatable and can cause gastrointestinal symptoms such as abdominal pain, diarrhea, and constipation in young children. These aspects are correlated with low dietary adherence that can expose patients to the risk of micronutrient deficiency. Maternal phenylketonuria (MPKU) poses risks to the developing fetus as elevated phenylalanine (Phe) levels, crossing the placental blood membrane, have well-recognized teratogenic effects [47]. This syndrome is characterized by intrauterine growth retardation, facial dysmorphism, microcephaly, congenital heart disease, low birth weight, developmental delay, intellectual disabilities, and an elevated risk of miscarriage, often linked to inadequate maternal metabolic control.

Maintaining a strict, phenylalanine-restricted diet is vital in maternal PKU to prevent harm to the fetus [45••, 46, 47]. Particular attention should be paid to dietary assessment, anthropometric parameters, and clinical features of micronutrient deficiency during every outpatient evaluation [46]. In addition to PKU, many other inherited metabolic diseases require a daily micronutrient supplementation, including maple syrup urine disease, isovaleric acidemia, methylmalonic aciduria, propionic aciduria, glutaric aciduria type 1, homocystinuria, hypermethioninemia, glycogen storage diseases, urea cycle disorders, fatty acids oxidation defects, and other rare diseases.

#### Cystic Fibrosis

Cystic fibrosis is the most common autosomal recessive disease reducing life expectancy [48]. The malfunctioning of the CFTR channel causes an accumulation of thickened mucus secretions in organs throughout the body, including the lungs, liver, pancreas, gallbladder, and intestines. Cystic fibrosis patients are prone to malnutrition and micronutrient deficiency primarily because the thickened secretions obstruct the intra-pancreatic ducts, reducing the delivery of digestive enzymes to the intestines and impairing the absorption of key nutrients. Exocrine pancreas insufficiency leads to poor absorption of fat-soluble vitamins. The severity of the CFTR variant correlates with pancreatic insufficiency: patients with severe variants (classes I, II, and III) have an altered exocrine function early in life, often at birth [49••]. Cholestasis and CFLD (cystic fibrosis-related liver disease) reduces available bile salts, and the lack of pancreatic bicarbonate secretions causes intestinal acidification, which further limits fatty acid and vitamin absorption. Moreover, these patients have an intrinsically higher metabolic demand, higher essential fatty acid turnover, and intestinal dysmotility [66]. Survival and lung function in children and adults inversely correlates with the degree of malnutrition [50]. As a consequence, nutritional care is a fundamental part of the multidisciplinary approach for these patients. Due to malabsorption and energy deficit, the standard of care for patients with cystic fibrosis is a high-calorie and high-fat diet with pancreatic enzyme replacement therapy (PERT) and the oral supplementation of vitamins [49••]. A daily supplementation of liposoluble vitamins A, D, K, and E is recommended [51]. Due to the increased sweating, intestinal malabsorption, and chronic inflammation, patients with cystic fibrosis may have higher than normal requirements for electrolytes and minerals: ESPGHAN recommends that sodium, iron, calcium, and zinc status be assessed regularly and supplemented if needed [51]. A strict nutritional assessment through evaluation of weight-for-length curves, longitudinal growth trajectory, and BMI is crucial to determine when to intensify the nutrition intervention. Moreover, it seems that antioxidants can positively impact disease outcome [52••]. The nutritional state of cystic fibrosis patients has significantly improved in recent years and is mainly attributed to early diagnosis through newborn screening.

### Developmental Conditions: Preterm and Low-Birth-Weight Infants

Preterm and low-birth-weight infants (SGA, small for gestational age) are intrinsically at risk of micronutrient deficiencies during the first 1000 days of life because of limited body stores, immature regulation systems, and high nutritional demands [53•]. Moreover, the third trimester of pregnancy is a period of rapid fetal brain growth and development in terms of cortical thickening, myelination, axonal development, vascularization, and cerebellar growth [54]. Fetal malnutrition, whether in excess or deficiency, can predispose to the development of chronic pathologies in adulthood. “Fetal programming” comprises the endocrine-metabolic conditions occurring during intrauterine life, which could contribute to long-term diseases through epigenetic mechanisms. Diet is a powerful modulator of epigenetics both in prenatal and post-natal life; it can condition DNA methylation and influence “fetal programming” [1]. IUGR (intra-uterine-growth-restriction) fetuses, due to maternal malnutrition, are at greater risk of developing impairment of metabolic and cardiovascular systems (hypertension, diabetes, glucose intolerance, insulin resistance). Pregnant women should be adequately informed of the importance of fetal nutrition on the health of the unborn child, motivating them to adopt a healthy lifestyle [55]. No evidence supports the improvement of fetal growth after nutrient supplementation by the pregnant mother in this specific circumstance [56]. Other factors influencing complementary food introduction in preterm/low birth weight infants are feeding difficulties and increased rate of acute illnesses. Limited agreement exists on nutritional management aimed at guaranteeing optimal growth and neurodevelopment [57••]. As a consequence, the length and dosage of micronutrient supplementation during the first 1000 days of life differ among centers. Peculiar micronutrient deficiencies in this patient category include iron, vitamin D, zinc, LCPUFAs, calcium, and phosphorus. Prematurity ceases placental iron transfer, and the following rapid catch-up growth typical of premature babies further reduces the body’s iron stores. AAP and ESPGHAN recommend tailored iron supplementation according to birth weight, gestational age, type of feeding, and iron status, with measurements of iron storage upon hospital discharge, during follow-up and at the beginning of complementary feeding [38••, 58]. The suggested dietary iron intake is 3–4 (max 6) mg/kg/die for VLBW infants (< 1500 g), 2–3 mg/kg/die for infants with birth weight < 1500 g, 2 mg/kg/die for weight 1500–2000 g, and 1–2 mg/kg/day for weight > 2500. Formula feeding provides 2.25 mg/kg of iron if consumed at 150 ml/kg/die; exclusive human feeding provides lower amounts [38••]. Although there is a lack of trials with long-term neuro-developmental outcomes, long-term iron supplementation appears to result in a reduction in iron deficiency and anemia in preterm and LBW infants [59]. As a consequence, in order to meet nutritional needs during the first 1000 days of life, iron supplementation should be continued in preterm/low birth weight infants after hospital discharge, with doses tailored according to iron status during complementary feeding and proper avoidance of iron overload [57••]. Because they lack an appropriate storage system, preterm newborns at 40 weeks post-conceptional age have lower serum zinc levels compared to full-term neonates. Even though serial measurement of zinc serum concentration is not recommended unless, in case of evidence of zinc deficiency (e.g., acrodermatitis enterohepatica or poor growth), zinc supplementation during the first year of life may be advisable, especially in children with impaired growth. The risk of zinc overload should not be a concern since it has no pro-oxidant effect (recommended daily intake of 2–2.25/kg, up to 3 mg/kg in extremely preterm neonates) [38••, 57••, 60–62]. Premature infants are at risk of osteopenia because most bone mineralization occurs during the third trimester of pregnancy, and postnatal calcium absorption is suboptimal. Fortification of breast milk or use of preterm formulas are recommended in hospitals, but there is no consensus on their use after discharge [38••, 57••, 63•, 64]. Assessment of metabolic bone biomarkers (vitamin D, calcium, phosphorus, alkaline phosphatase (ALP), parathyroid hormone) is advisable in VLBW infants 2–4 weeks after discharge [65]; oral calcium and phosphate supplementation should be started when ALP levels are equal or above 800–1000 UI/ml [65] with a total daily intake of 120–200 mg/kg/die and 70–115 mg/kg/die respectively (ESPHGHAN 2022) [38••]. Neonatal vitamin D storage is directly related to maternal vitamin D status. A sufficient vitamin D supply is difficult to achieve even for full-term newborns because both human and formula milk contain inadequate levels; rapid growth and long hospitalization of preterm infants can further increase the risk of deficiency. Hence, vitamin D prophylaxis should be implemented in all infants for the first year of life at the dosage of 400 UI/die; ESPGHAN suggests a higher dose for preterm infants up to full-term corrected age (daily vitamin D intake of 400–700 IU/kg/die, maximum dosage 1000 IU/die) [21•, 22, 38••, 57••, 66]. Placental transfer of LCPUFA (DHA (docosahexaenoic acid) and AA (arachidonic acid)) occurs mostly during the third trimester, and brain accumulation is considerable. Moreover, preterm infants are unable to convert fatty acids precursors (linoleic acid (LA) and α-linolenic acid (ALA)) to DHA due to high requirements of fatty acids and metabolic immaturity. As conflicting results derive from studies investigating the benefits of LCPUFA supplementation, there is currently no sufficient evidence for consensus recommendation [38••, 57••]. Preterm infants fed orally could be at risk for folate deficiency. Modern preterm formulas have decreased the need for folic acid supplementation, although folic acid supplementation remains common [67•].

### Behavioral Conditions: Attention-Deficit/Hyperactivity and Autism Spectrum Disorders

Nutritional deficiencies and quality of the diet are linked to the pathogenesis of various common mental disorders, such as depression, schizophrenia, autism spectrum disorder, and attention-deficit hyperactivity disorder. Specifically, diet can interact with other lifestyle factors, and children with attention-deficit/hyperactivity disorder (ADHD) show less adherence to healthy eating patterns than children without this disorder [68••, 69]. Consequently, low blood levels of zinc, magnesium, and ferritin are detected in children with ADHD [70•]. Feeding problems, such as food selectivity, food refusal, and abnormal dietary patterns, are highly prevalent in children with autistic spectrum disorder [71, 72]. Food selectivity causes a reduced consumption of whole grains, milk and dairy products, beans and soy products, vegetables, and fruits by children with autism spectrum disorder. When comparing the nutritional status of children with autism with that of neurotypical children, differences can be found in biomarkers indicative of vitamin insufficiency and increased oxidative stress, with several biomarkers associated with variations in the severity of autism. Studies report decreased dietary intake and lower serum levels in folate; vitamins B12, D, A, E, and K; iron; calcium; and zinc in children with autism compared to neurotypical controls [73]. Therefore, it is essential to provide detailed nutritional evaluation and individualized nutrition interventions for children with ADHD and autism spectrum disorders.

### Acquired Conditions

#### Epilepsy Requiring a Ketogenic Diet

Ketogenic dietary therapies (KDT) are considered an adjuvant treatment in several medical conditions, included drug-resistant forms of epilepsy but also inherited metabolic disorders like GLUT1 deficiency syndrome and pyruvate dehydrogenase deficiency, Prader-Willi syndrome, and some specific types of cancers. Exploiting a process known as nutritional ketosis, the diet reduction of carbohydrates intake facilitates ketogenesis in order to provide an alternate source of energy. It has proved to reduce the frequency and severity of seizure in some forms of epilepsy [74•]. The ketogenic diet also poses some issues as nausea, constipation, fatigue, dehydration, electrolyte imbalances, and micronutrient deficiency. The limited intake of fruits, vegetables, carbohydrates, and calcium-rich foods can lead to vitamin and mineral deficiencies. In children following KDT, some common deficiencies include vitamin B, vitamin C, vitamin D, calcium, selenium, and magnesium [75] due to limited intake of grains, fruit and vegetables. KDT are individualized treatments carefully planned by multidisciplinary teams. Multivitamin and mineral supplements that are free of carbohydrates are always recommended to prevent micronutrient deficiencies [76, 77]. Constant monitoring is necessary for all patients on KDT in order to assess its impact on child’s growth. Further studies are needed to investigate possible long-term effects of these diets when started in children [74•].

#### Food Allergies

The diet of expectant mothers can induce tolerance to solid food in the offspring through the influence of fetal immune development [78•], even though, at present, it is not clear whether any one nutrient is more important in terms of prevention [79]. Nonetheless, antigen avoidance by expecting and breastfeeding mothers is not suggested to prevent allergy, even in high-risk children [78•, 79–81]. Certain foods are more common triggers of significant acute allergic reactions during the first 1000 days of life, i.e., cow’s milk, hen’s egg, soy, wheat, peanuts, and seafood. Children with a familiar or personal history of atopy are at higher risk of developing other atopic diseases. Currently, a delay in the introduction of complementary foods in the diet of high-risk infants beyond what is generally recommended for all infants (from the 5–6th month) is not recommended. No skin prick or IgE testing screening is necessary before introducing the allergen [82••]. Risk factors for nutritional deficits in patients undergoing elimination diets during the first 1000 days of life are represented by multiple food allergies, altered weaning schedules, elimination of food staples such as cow’s milk or wheat, and picky eating. All allergic patients on elimination diets would benefit from referral to a dietitian to learn how to substitute the eliminated food while minimizing the risk of nutrient deficiencies and poor growth [79, 83, 84••, 85••].Wheat is a major source of iron, thiamine, niacin, riboflavin, folic acid, magnesium, and vitamin B6; choosing whole or enriched available alternative grains improves the nutritional quality of a wheat-restricted diet during the first 1000 days of life [86]. Eggs contain vitamin B12, riboflavin, biotin, and selenium, and they are common ingredients in the Western diet; therefore, allergic patients need to learn to replace eggs in recipes [87••]. Egg replacement products, which can be used in cooking and baking, are available on the market, and although they provide a similar consistency, they are not similar to eggs in terms of nutritional profile. However, the micronutrients contained in eggs can be found in a wide range of other animal products. Soybean, peanuts, tree nuts, and shellfish elimination, if not combined with allergies to previously mentioned food, is unlikely to impact nutritional intake [79] negatively. Most individuals with soy allergy can tolerate highly refined soy oil and soy lecithin, which are present in many manufactured foods. Fish has an important nutritional value; it is rich in vitamins B, D, and A; selenium; calcium and phosphorus; iron; zinc; magnesium; iodine; and omega-3. Since Omega-3 is present in a few other foods, supplementation could be advisable in children affected by fish allergy [88]. However, because individuals with seafood allergy are rarely allergic to all seafood, people with fish allergy may be able to eat shellfish, and those allergic to shellfish may tolerate canned fish [89•]. Supplements may be necessary for individuals with multiple food allergies, especially those excluding other foods in addition to milk. Table 2 summarizes micronutrients provided from excluded foods and possible substitute.

#### Celiac Disease

Celiac disease is a hereditary disorder caused by sensitivity to the gliadin fraction of gluten, a protein contained in wheat, rye, and barley. In a genetically susceptible individual, gluten-sensitive T-cells are activated upon contact with gluten; this causes an inflammatory response in the small bowel, leading to the mucosal villi’s characteristic atrophy. Symptoms in children include failure to thrive and non-specific gastrointestinal manifestations. Laboratory abnormalities often occur and include anemia due to malabsorption, namely, iron deficiency or folate deficiency anemia. Because the iron absorption process develops mainly in the proximal duodenum, iron deficiency anemia is frequently the presenting symptom of the disease in children. International recommendations underline the importance of periodic screening for essential micronutrient deficiency in celiac patients, focusing on iron, folic acid, and vitamins D and B12. The treatment of celiac disease consists of a gluten-free diet (avoiding foods containing wheat, rye, and barley) aimed at reducing the inflammatory response and restoring the functional anatomy of intestinal villi, along with vitamin and mineral supplements depending on the type of deficiency [90, 91].

## Nutrition-Drug Interaction

Prolonged intake of some drugs can cause micronutrient deficiencies due to decreased absorption or increased gastrointestinal loss. Examples of drugs used in the first 1000 days which could interfere with micronutrient absorption are reported in Table 3. The main action of proton pump inhibitors (PPIs) is to reduce the production of gastric acid; therefore, their use can lead to a deficiency in micronutrients whose absorption depends on low gastric pH. Gastric acid also plays a key role in the intestinal absorption of B12 from food proteins. PPI use reduces the absorption of protein-bound B12 and may lead to B12 deficiency in some individuals, although results are conflicting. There is insufficient evidence to recommend routine screening of vitamin B12 status or routine supplementation of patients taking PPIs [92, 93]. Moreover, some evidence indicates that PPI use may harm iron, calcium and vitamin C absorption. Nonetheless, there is no evidence of duration or dose–response effect, and the possibility of existing cofactors cannot be excluded [94]. Antibiotics alter the intestinal bacterial flora, decreasing the synthesis of folic acid and vitamin K. Diuretics cause renal loss of vitamins (group B, especially B1, and vitamin C) and minerals (especially potassium, magnesium and calcium). Bile acids sequestrant resins can reduce the absorption of fat-soluble vitamins. Aspirin can cause a significant reduction in vitamin C in white blood cells and blood platelets, with consequent bleeding risks. Nonsteroidal anti-inflammatory drugs (NSAIDs) and corticosteroids can also reduce the availability of calcium, magnesium, folic acid, potassium, vitamin C, vitamin D, and selenium. Some chemotherapeutic drugs act by inhibiting the transformation of folic acid into its active form, blocking a fundamental process for cell replication. Antitubercular drug isoniazid cause vitamin B6 deficiencies. Antiepileptics, such as phenobarbital and phenytoin, alter the absorption of vitamins and calcium.

**Table 3:** Most commonly used drugs potentially causing micronutrient deficiency in the first 1000 days

Drug/drug class	Potential micronutrient deficit
Proton pump inhibitors (PPIs)	B12, iron, calcium, vitamin C
Antibiotics	Folate, vitamin K
Isoniazide	Vitamin B6
Antiepileptic drugs (phenobarbital and phenytoin)	Biotin, folate, vitamin B6, vitamin D, vitamin K
Antipsychotic drugs	Vitamin B2, vitamin D
Methotrexate	Folate
Bile acids sequestrant resins	Vitamin A, vitamin D, vitamin E, vitamin K
Corticosteroids and NSAIDs	Calcium, magnesium, folic acid, potassium, vitamins C and D, and selenium
Rifampicin	Vitamin D, vitamin K
Sulfa-drugs	Folate
Diuretics	Vitamin B, vitamin C, potassium, magnesium, and calcium

## Conclusions

Early life malnutrition in terms of both macro and micronutrients can cause metabolic derangements which could impair or delay the physical and cognitive development of the individual. Deficiencies should be promptly identified in order to tailor interventional strategies specifically to the metabolic needs of the individual [95, 96]. When dietary reference intakes are unmet, collaboration between the pediatrician and a pediatric nutritionist or dietitian is advisable. Nutritional biomarkers are useful for assessing nutrient intake, but they have limitations in terms of accuracy. Just combining anamnesis, clinical evaluation, growth, and dietary assessment, pediatricians can effectively identify and address potential micronutrient deficiencies in children during the first 1000 days of life. Overall, most micronutrient deficiencies can be prevented during the first 1000 days of life through nutrition guidance, food fortification, or supplementation. Timely supplementation can provide a lifelong advantageous impact on child development. It is evident that pediatricians have a fundamental task. However, both environmental factors and a cultural worsening of dietary habits make it difficult. On one hand, micronutrient content can be altered by rising temperature and increasing atmospheric carbon dioxide, reducing the overall yield of micronutrient-rich foods (fruits, vegetables, fish, and nuts). On the other hand, consumption of excessive quantities of refined and processed foods and sugar-sweetened beverages is increasing, contributing to inadequate intake of micronutrients [77]. In conclusion, nutrition in the first 1000 days of life through correct, specific, and precise guidance could help prevent the majority of any cardio-vascular risk factors over time.

### Supplementary Information

Below is the link to the electronic supplementary material.

Supplementary file1 (DOCX 23 KB)

## Data Availability

The data that support the article are available on request from the corresponding author.
