# Supplementary material for: Potential Micronutrient Deficiencies in the First 1000 Days of Life: The Pediatrician on the Side of the Weakest
Source: Curr Obes Rep. 2024 Mar 21;13(2):338–51. doi: 10.1007/s13679-024-00554-3 (PMC11150320; doi:10.1007/s13679-024-00554-3)
Supplement: Supplementary file 1 — Supplementary file1 (DOCX 23 KB) [file 13679_2024_554_MOESM1_ESM.docx]

VISUAL ABSTRACT
